# Pioglitazone attenuates tamoxifen-induced liver damage in rats via modulating Keap1/Nrf2/HO-1 and SIRT1/Notch1 signaling pathways: *In-vivo* investigations, and molecular docking analysis

**DOI:** 10.1007/s11033-023-08847-x

**Published:** 2023-11-07

**Authors:** Gellan Alaa Mohamed Kamel, Hemat A. Elariny

**Affiliations:** Department of Pharmacology and Toxicology, Faculty of Pharmacy (Girls), Al-Azhar University, P.N. 11754, Nasr City, Cairo, Egypt

**Keywords:** Tamoxifen, Pioglitazone, Hepatotoxicity, Notch1, Sitruin1, Nrf2

## Abstract

**Background:**

Tamoxifen (TAM) is a chemotherapeutic drug widely utilized to treat breast cancer. On the other hand, it exerts deleterious cellular effects in clinical applications as an antineoplastic agent, such as liver damage and cirrhosis. TAM-induced hepatic toxicity is mainly attributed to oxidative stress and inflammation. Pioglitazone (PIO), a peroxisome proliferator-activated receptor-gamma (PPAR-γ) agonist, is utilized to treat diabetes mellitus type-2. PIO has been reported to exert anti-inflammatory and antioxidant effects in different tissues. This research assessed the impact of PIO against TAM-induced hepatic intoxication.

**Methods:**

Rats received PIO (10 mg/kg) and TAM (45 mg/kg) orally for 10 days.

**Results:**

TAM increased aspartate aminotransferase (AST) and alanine aminotransferase (ALT), triggered several histopathological alterations, NF-κB p65, increased hepatic oxidative stress, and pro-inflammatory cytokines. PIO protects against TAM-induced liver dysfunction, reduced malondialdehyde (MDA), and pro-inflammatory markers along with improved hepatic antioxidants. Moreover, PIO, increased hepatic Bcl-2 expression while reducing Bax expression and caspase-3 levels. In addition, PIO decreased Keap-1, Notch1, and Hes-1 while upregulated HO-1, Nrf2, and SIRT1. Molecular docking showed the binding affinity of PIO for Keap-1, NF-κB, and SIRT1.

**Conclusion:**

PIO mitigated TAM hepatotoxicity by decreasing apoptosis, inflammation, and oxidative stress. The protecting ability of PIO was accompanied by reducing Keap-1 and NF-κB and regulating Keap1/Nrf2/HO-1 and Sirt1/Notch1 signaling.

**Graphical abstract:**

A schematic diagram illustrating the protective effect of PIO against TAM hepatotoxicity. PIO prevented TAM-induced liver injury by regulating Nrf2/HO-1 and SIRT1/Notch1 signaling and mitigating oxidative stress, inflammation, and apoptosis.
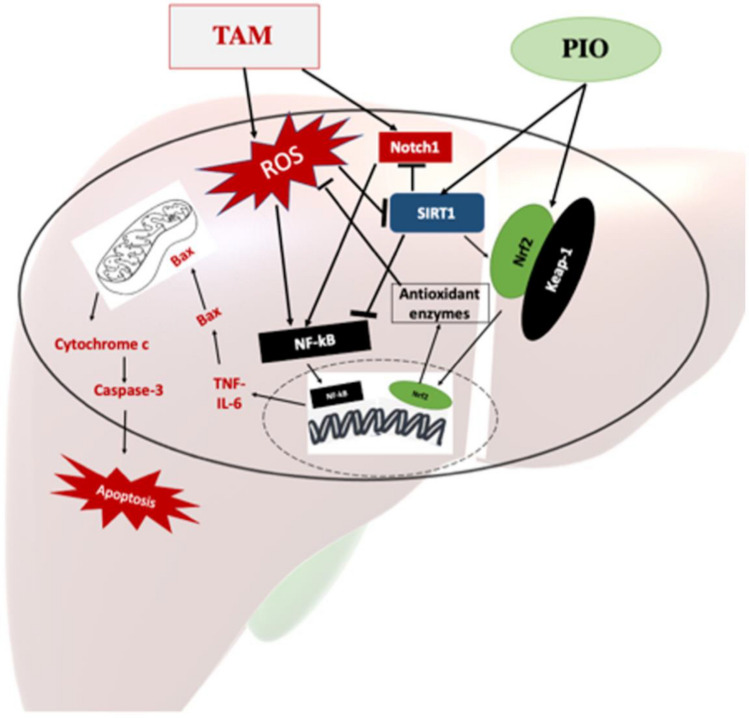

## Introduction

Tamoxifen (TAM) is an anti-estrogen non-steroidal agent utilized extensively as a chemotherapeutic drug to cure breast cancer [1]. However, several investigations indicated that TAM exerts detrimental effects in non-target tissues including liver and kidney organs [2–6]. TAM has been determined to induce apoptosis and tissue injury via stimulation of oxidative stress and inflammatory pathways [2, 3, 7]. TAM was documented to provoke reactive oxygen species (ROS) production via the enzymes of antioxidant impairment and raising lipid peroxidation [8, 9]. Nuclear factor-kappa B (NF-κB), which triggers the transcription of the inflammatory intermediates and causes inflammation and liver damage, is one of the signaling molecules that may be activated by ROS. Thus, hindering ROS production might control liver damage caused by TAM [3, 8–10].

It has been confirmed that nuclear factor (erythroid-derived 2)-like 2 (Nrf2) activation is essential for the defense against drug-induced liver damage [11–13]. In the cytosol, where it is sequestered by the Kelch-like ECH-associated protein 1 (Keap1), is the redox-sensitive transcription factor Nrf2, which is essential for healthy cell function. It separates and moves into the nucleus after being exposed to ROS, where it connects to the antioxidant response element (ARE) and activates the production of heme oxygenase (HO)-1 that has anti-inflammatory and antioxidant features [14].

Sirtuin-1 (SIRT1), one of the sirtuins family, has been linked to a number of cellular activities, including mitochondrial biogenesis, stress responses, and inflammation. It may deacetylate a variety of substrates [15–17]. Prior researches has suggested that SIRT1 controls downstream transcription parameters including NF-κB and Nrf2 to prevent oxidative stress and inflammation damage [18–20]. Besides, Notch1 signaling is noted as a downstream effector pathway of SIRT1, which is reported to control inflammatory reactions and redox status [16]. The Notch intracellular domain (NICD), when activated, causes the complex to switch from being a repressor to an activator of Notch target genes by trans-locating from the cytoplasm to the nucleus in which it interacts to C protein binding factor-1 (CSL). The hairy enhancer of split-1 (Hes-1) one of the downstream effector genes was documented to induce NF-κB gene transcription. Notch signaling is also regarded to have an essential effect on the pathophysiology of liver injury [21, 22]. Therefore, modulation of Nrf2 and SIRT1/Notch1 signaling can suppress TAM-induced hepatotoxicity by alleviating inflammation and oxidative stress.

Pioglitazone (PIO), a peroxisome proliferator-activated receptor-γ (PPAR-γ) agonist, is prescribed for the treatment of diabetes type 2 [23]. PPAR-γ modulates cellular proliferation, angiogenesis, and metastasis by suppressing the expression of NF-κB [24]. PIO has a broad spectrum of biological actions such as antioxidant, anti-inflammatory, neuroprotective, hepatoprotective, and other impacts [15, 25–27]. PIO has been documented to exert hepatoprotective effects against hepatotoxicity caused by different agents such as cyclophosphamide, and acetaminophen [28–30]. Yet, the method underlying the hepatoprotective influence of PIO is not completely clarified. This work studied the protecting role of PIO against TAM-induced liver damage indicating the contribution effect of Keap1/Nrf2/HO-1 and SIRT1/Notch signaling. Additionally, molecular docking modeling was accomplished **to** investigate the binding capability of PIO to Keep-1, NF-κB, and SIRT1.

## Materials and methods

### Chemicals and drugs

The chemicals were obtained from Sigma-Aldrich (St. Louis, MO, USA) and were of the highest analytical quality. TAM (Nolvadex^®^) tablets (10 mg) were obtained from AstraZeneca (Egypt). It was given orally at a dose of 45 mg/kg after being dissolved in Tween 80 (1%). PIO (Actos^®^) tablets (30 mg) were obtained from Hikma pharmaceuticals company (Egypt) and dissolved in saline.

### Animals

18 female albino rats weighing between 150 and 180 g were acquired from the EL-Nile Co. for Pharmaceuticals and Chemical Industries’ animal facility in Cairo, Egypt. The experimental animals were kept in a controlled environment with a humidity level of 55%, a temperature range of 20 to 25 °C, and a light/dark cycle of 12 h, as well as frequent feedings and unlimited access to water. Prior to any experimental technique, animals had a one-week acclimatization period. Following the recommendations of the Animal Ethics Committee of the Faculty of Pharmacy, Al-Azhar University, Egypt (Number: 387), the experiments were carried out.

### Experimental design

Rats were randomly divided into three groups (n = 6 rats in each group).

1. Control group; rats received 1% Tween 80 for 10 days orally by oral gavage.
2. TAM group: rats administrated TAM (45 mg/kg) for 10 days orally by oral gavage [5, 7].
3. PIO + TAM group: rats received PIO (10 mg/kg) orally by oral gavage one hour before TAM administration and continued for 10 consecutive days. PIO dose was chosen regarding to prior researches [31, 32]. Blood samples were taken via every animal’s retro-orbital vein at the completion of the experiment while they were anesthetized (ketamine 75 mg/kg and xylazine 10 mg/kg, i.p.). To achieve clear sera, blood specimens were centrifuged at 1000 × g for 15 min after being allowed to clot. Anesthetized rats were decapitated and immediately slaughtered after blood samples were taken. The livers were then taken out, washed with normal saline, and weighed. While other liver tissue specimens were homogenized in cold PBS (10% w/v), fixed in 10% neutral-buffered formalin (NBF), centrifuged, and the clear supernatant was obtained for examination. For RNA isolation, additional samples were obtained on RNA later and kept at 80 °C.

### Determination of liver function markers

Rat serum was assessed for alanine aminotransferase (ALT), aspartate aminotransferase (AST), and total bilirubin levels utilizing colorimetric kits (Abcam, UK), according to manufacturer instructions.

### Histopathological examination

After being fixed in 10% NBF for 24 h, the liver samples were dehydrated in increasing concentrations of ethanol, cleaned in xylene, and then embedded in paraffin wax. Utilizing a microtome, 5-μm slices were cut, and stained with hematoxylin and eosin (H&E) [33].

### Assessment of the hepatic oxidative stress markers (lipid peroxides, SOD, GPx, and GSH) and HO-1 levels

Lipid peroxidation assay was performed in hepatic homogenate by measurement of malonaldehyde (MDA) content utilizing a commercial kit (Bio-diagnostic Co., Egypt). Glutathione peroxidase (GPx) and Superoxide dismutase (SOD) activities along with glutathione (GSH) levels were carried out utilizing kits obtained from (Bio-diagnostic Co., Egypt). For the determination of HO-1 level, an ELISA kit was collected from (My BioSource Inc, USA). The procedures were conducted in accordance with the manufacturer’s strategy, and the parameters were chosen from those that the manufacturer had specified.

### Determination of inflammatory cytokines

IL-6 and TNF-α pro-inflammatory cytokines were quantified using an ELISA kit from (Cloud-clone Corp., USA) and (RandD Systems, Inc., USA), respectively. The manufacturer’s methodology was strictly adhered to in accordance with the guidelines for every factor that was evaluated, and concentration samples were taken according to the instructions of the manufacturer.

### Quantitative real-time polymerase chain reaction (qRT-PCR) analysis

Real-time PCR was utilized to ascertain the expression levels of mRNA for NF-κB p65, Nrf2, Keap1, SIRT1, Notch1, Hes-1, Bax, and Bcl-2 utilizing Step One TM System (Applied Biosystems). Whole RNA was isolated utilizing QIAzole and Qiagen RNeasy Mini Kits (Qiagen, Hilden, Germany). The Nanodrop (ND-1000, USA) was used to confirm that the RNA was both pure and high in concentration. The primers from (Thermofisher, USA) used for evaluations are listed in Table 1. Utilizing SYBR Green master mix from Thermofisher in the United States, quantitative real-time one-step PCR was carried out. After a 10-min hot start at 95 °C, samples were exposed to 45 cycles of 1 min of annealing at 54–58 °C and 15 s of denaturation at 95 °C. To check for non-specific products, melting curve analysis was carried out from 60 °C up to 95 °C with moderate temperature increments of 0.5 °C every 10 s. The Ct value, which denotes the cycle number once the fluorescence curve passed the baseline value, was calculated utilizing fluorescent quantitative analysis utilizing the thermal cycler’s (Veriti^®^) software package to assess the ΔCt value. The 2^−ΔΔCt^ technique was utilized to normalize the target genes’ relative mRNA expression levels to those of the control group and the concentration of β-actin.

**Table 1:** Primer sequences used in real-time RT-PCR analysis

Gene	Forward primers (5′–3′)	Reverse primers (5′–3′)
Nrf2	TTGTAGATGACCATGAGTCGC	TGTCCTGCTGTATGCTGCTT
Keap1	TCCTCAGAGGGCAGTGGAAT	TATGTGTCCCACAAGGGAGC
SIRT1	TCTCCCAGATCCTCAAGCCAT	TTCCACTGCACAGGCACATA
NF-κB p65	TGGGACGACACCTCTACACA	GGAGCTCATCTCATAGTTGTCC
Notch1	ACAGTGCAACCCCCTGTATG	CGCAGGAAGTGGAAGGAGTT
Hes1	CTACCCCAGCCAGTGTCAAC	AATGCCGGGAGCTATCTTTCT
Bax	AGGACGCATCCACCAAGAAG	CAGTTGAAGTTGCCGTCTGC
Bcl-2	ACTCTTCAGGGATGGGGTGA	TGACATCTCCCTGTTGACGC
β-Actin	GGAGATTACTGCCCTGGCTCCTAGC	GGCCGGACTCATCGTACTCCTGCTT

### In silico molecular docking analysis

In silico molecular docking simulations were used to simulate the molecular interactions of PIO with (PDB ID: 4L7B), NF-κBp50.p65 (PDB ID: 1LE9)) and SIRT1 (PDB ID: 4ZZJ), as mentioned previously [14, 34, 35]. The protein data bank (PDB) was used to find the 3D crystal structures of the proteins. All proteins and PIO were prepared, and energy was minimized by the MMFF94 force field. The molecular docking was done, thirty poses were generated then the best orientations were captured, and 3D and 2D figures were generated by Biovia Discovery Studio 2019 Visualizer [36].

#### Statistics

Means ± SEM are used to present the data. Data were statistically analyzed utilizing one-way ANOVA and the post hoc Tukey’s multiple comparison test utilizing GraphPad Prism software (version 5). Values of P < 0.05 were considered statistically significant for the analyses.

## Results

### PIO treatment ameliorates TAM-induced liver injury

**Fig. 1:**
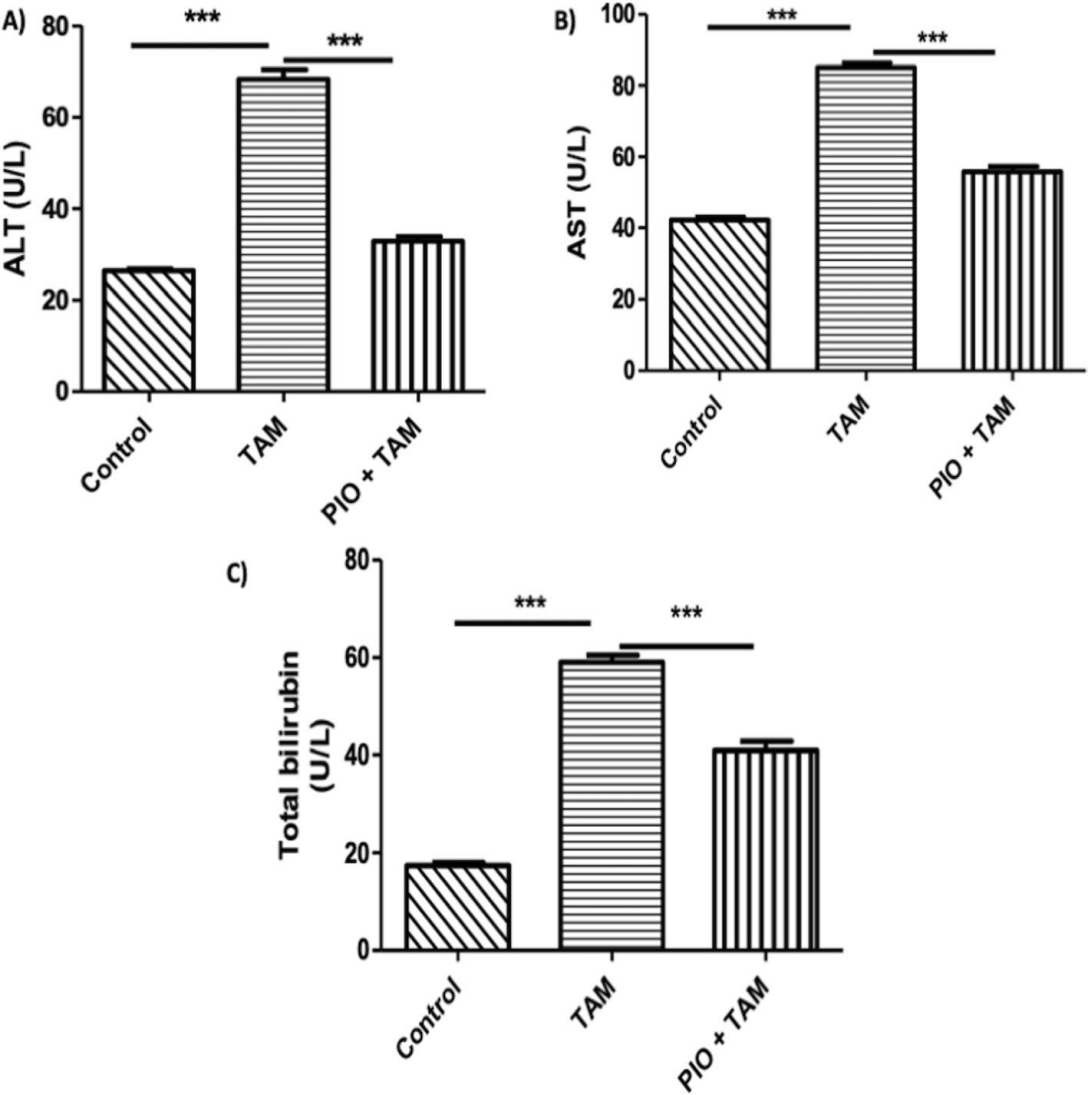
PIO attenuated TAM-induced liver damage in rats. PIO reduced serum ALT (**A**), AST (**B**), and Total bilirubin (**C**) in TAM-receiving rats. Data are mean ± SEM, n = 6. *P < 0.05, **P < 0.01, and ***P < 0.001

**Fig. 2:**
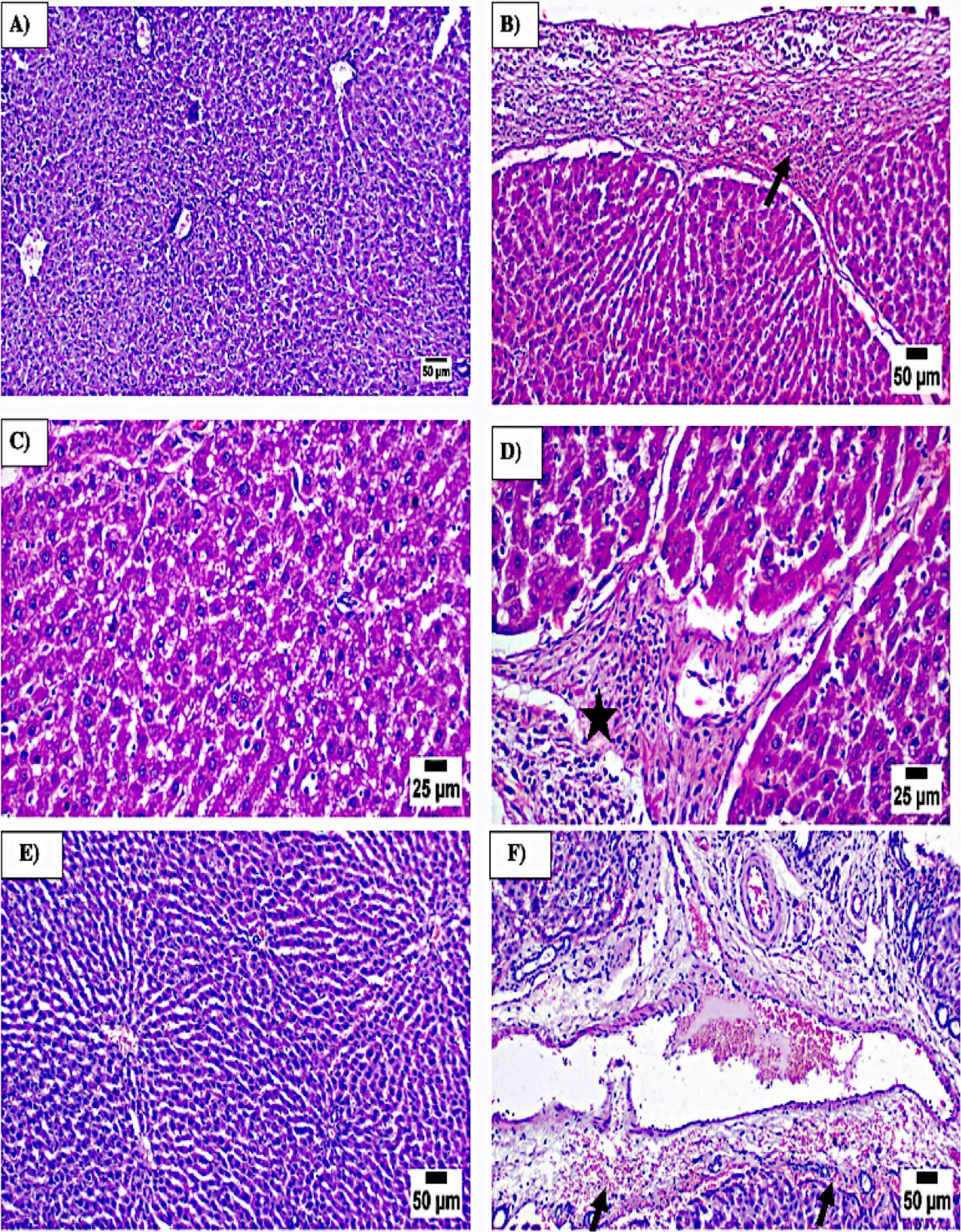
PIO alleviated TAM-induced liver histopathological alterations in rats. Photomicrographs of sections in the liver of **A** control rats revealing normal hepatic lobule structure and normal hepatocytes, **B–D** TAM rats showed caused tissue injury manifested by **B** thickened hepatic capsule with inflammatory cells infiltration (arrow), **C** extensive hepatocellular vacuolation, **D** portal fibroplasia (star) with mononuclear inflammatory cells infiltration, **E, F** PIO-treated portal (**E**) typical hepatocyte and hepatic lobule anatomy (**F**) edema and hemorrhage (arrow).

To evaluate the preventive effectiveness of PIO against TAM hepatotoxicity, liver function indicators in the serum were identified, and a histological investigation was carried out. Following TAM injection, there was an increase in serum ALT (Fig. 1A), AST (Fig. 1B), and total bilirubin (Fig. 1C) (P < 0.001). In contrast, PIO dramatically decreased the increased blood levels of AST, ALT, and total bilirubin in contrast to rats receiving TAM. The Microscopic analysis demonstrated normal hepatic lobule anatomy and normal hepatocytes in control samples, supporting the biochemical results (Fig. 2A). TAM caused tissue injury manifested by thickened hepatic capsule with inflammatory cells infiltration (arrow) (Fig. 2B), extensive hepatocellular vacuolation (Fig. 2C), portal fibroplasia (star) (Fig. 2D) with mononuclear inflammatory cells infiltration. However, PIO treatment improved hepatic histopathological alterations in TAM-treated rats (Fig. 2E), and only portal edema and hemorrhage were displayed in some areas (arrow) (Fig. 2F).

### PIO treatment attenuates TAM-induced hepatic oxidative stress in TAM-administered rats

**Fig. 3:**
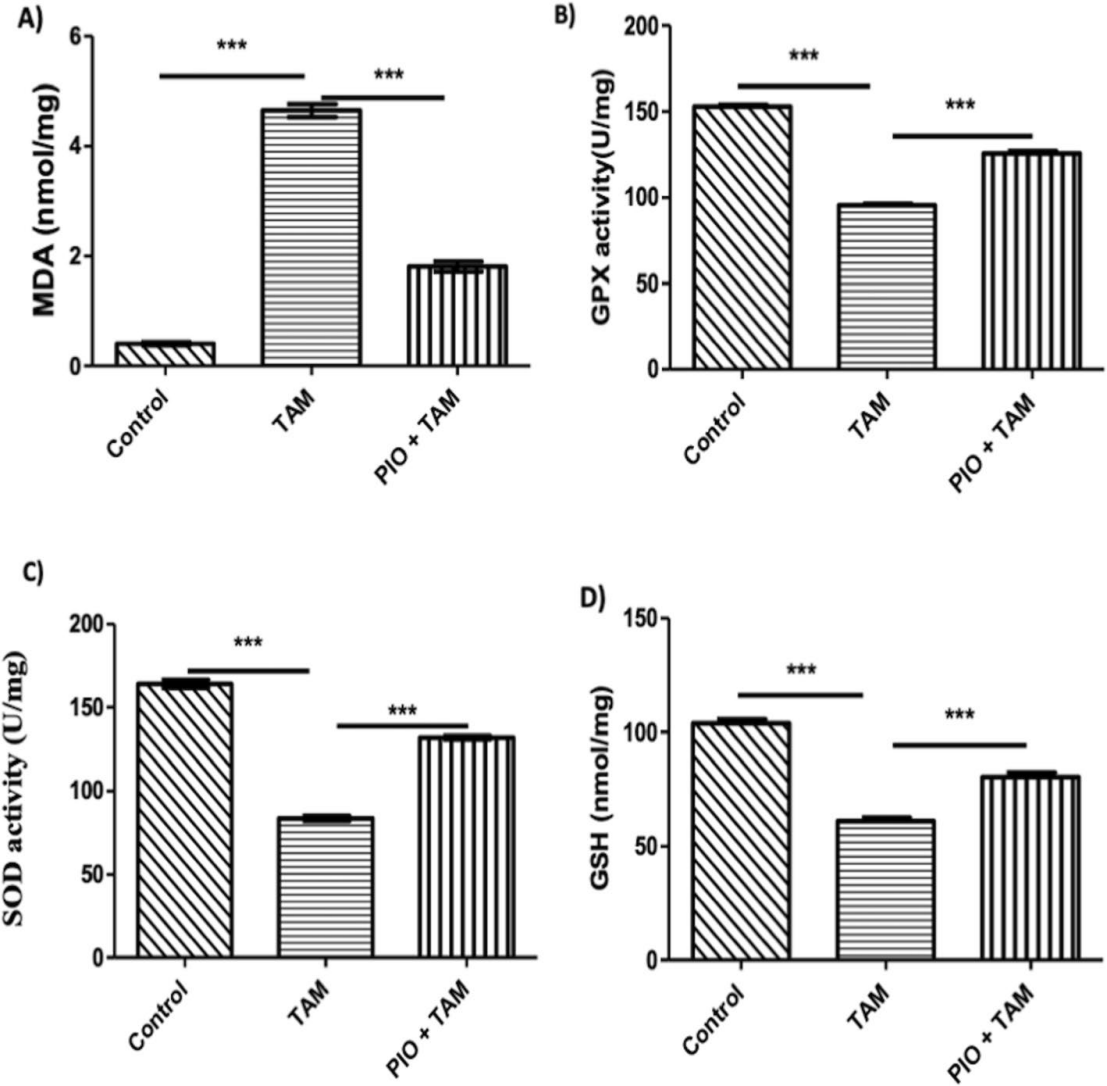
PIO attenuated TAM-induced hepatic oxidative stress. TAM reduced liver MDA (A) and raised GPX (B), SOD (C), and GSH (D) in TAM-administered rats. Data are mean ± SEM, n = 6. ***P < 0.001. *TAM* tamoxifen, *MDA* malonaldehyde, *PIO* pioglitazone, *GSH* glutathione, *GPx* glutathione peroxidase, *SOD* superoxide dismutase

To ascertain the impact of TAM on the redox state and the protective function of PIO, MDA, and antioxidants were evaluated. MDA (Fig. 3A) was raised in the liver of rats that administrated TAM (P < 0.001). GPx (Fig. 3B), SOD (Fig. 3C), and GSH (Fig. 3D) were reduced in TAM-treated rats (P < 0.001). PIO decreased MDA and as well as increased GPx, SOD, and GSH significantly compared to TAM-administered rats.

### PIO treatment mitigates hepatic inflammation in TAM-administered rats

**Fig. 4:**
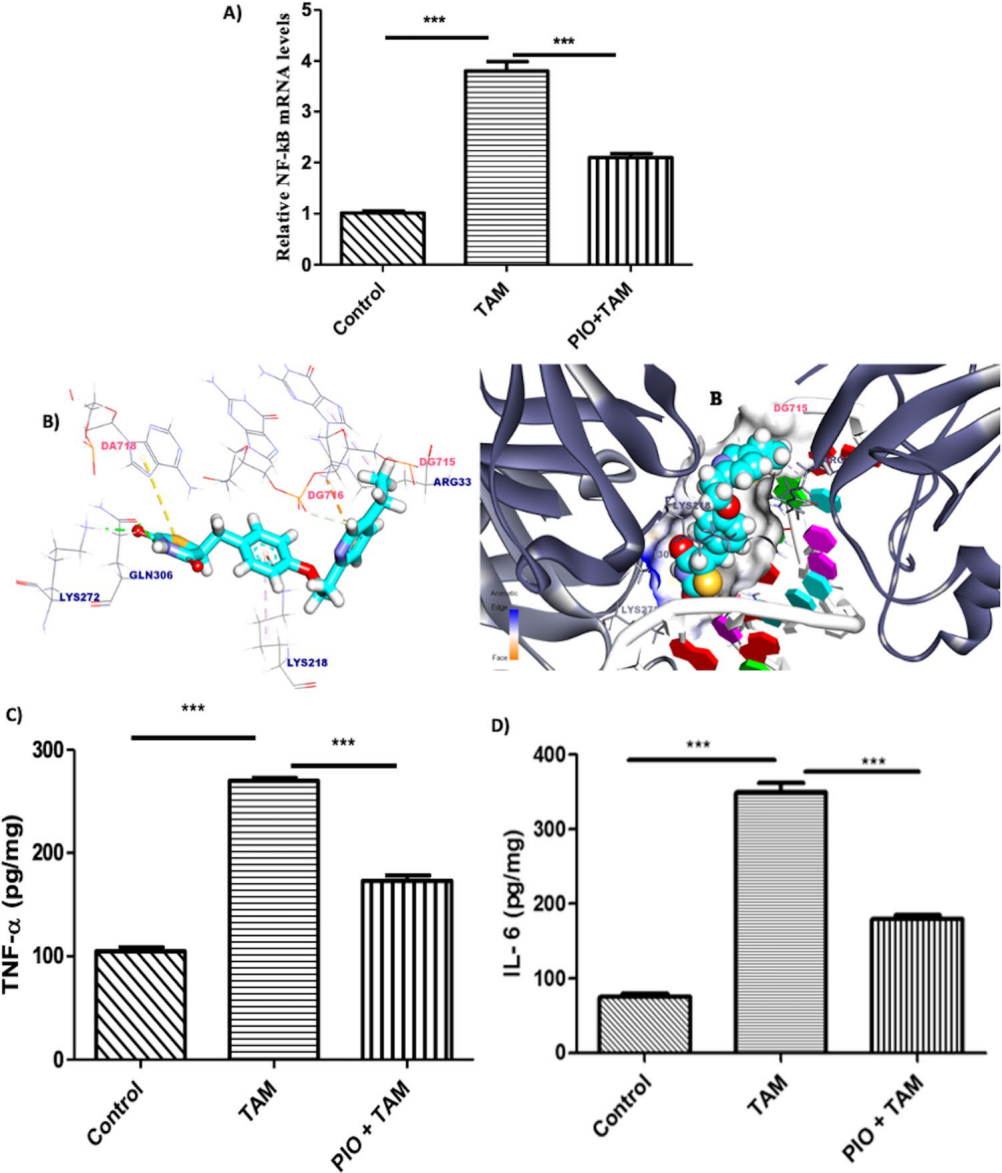
PIO inhibited hepatic inflammation in TAM-administered rats. PIO reduced NF-κB mRNA levels (**A, B**) molecular docking study showed that PIO binds to the NF-κBp50.p65 heterodimer. PIO binds with residues Lys272 and Gln306 to establish a hydrogen bond in the binding pocket of NF-κB, which is surrounded by DA718, DG715, Lys218, and Arg33, and is located there. Additionally, PIO reduced the levels of TNF-α (**C**) and IL-6 (**D**) in the liver of rats given TAM. Data are presented as the mean ± standard error of the mean (n = 6). *P < 0.05, **P < 0.01, and ***P < 0.001. *TAM* tamoxifen, *PIO* pioglitazone, *IL-6* interleukin-6, *NF-κB* nuclear factor kappa B, *TNF-α* tumor necrosis factor-alpha

As displayed in Fig. 4A, TAM considerably (P < 0.001) raised NF-κB p65 mRNA in the rats liver. In rats given TAM, PIO dramatically reduced hepatic NF-κB p65. Through the use of molecular docking, the NF-κB suppressive impact of PIO was further assessed. Figure 4B illustrates the in-silico docking profile for PIO’s interaction with the NF-κBp50.p65 heterodimer. PIO was positioned in the binding pocket of NF-κB by five Pi–sulfur, Pi–Alkyl, and Pi–cation interactions with nucleotide DA718, DG715, Arg33, and Lys218, and created two hydrogen bonds bond with the residues Lys272 and Gln306 with a bond length of 2.14 and 2.17 Å. According to the scoring function result (− 7.48 kcal/mol), it was determined that PIO had a strong binding to NF-κB. The findings demonstrating a considerable reduction in liver TNF-α (Fig. 4C) and IL-6 (Fig. 4D) in TAM-administered rats co-treated with PIO further validated the anti-inflammatory activity of PIO.

### PIO treatment reduced hepatic apoptosis in TAM-administered rats

**Fig. 5:**
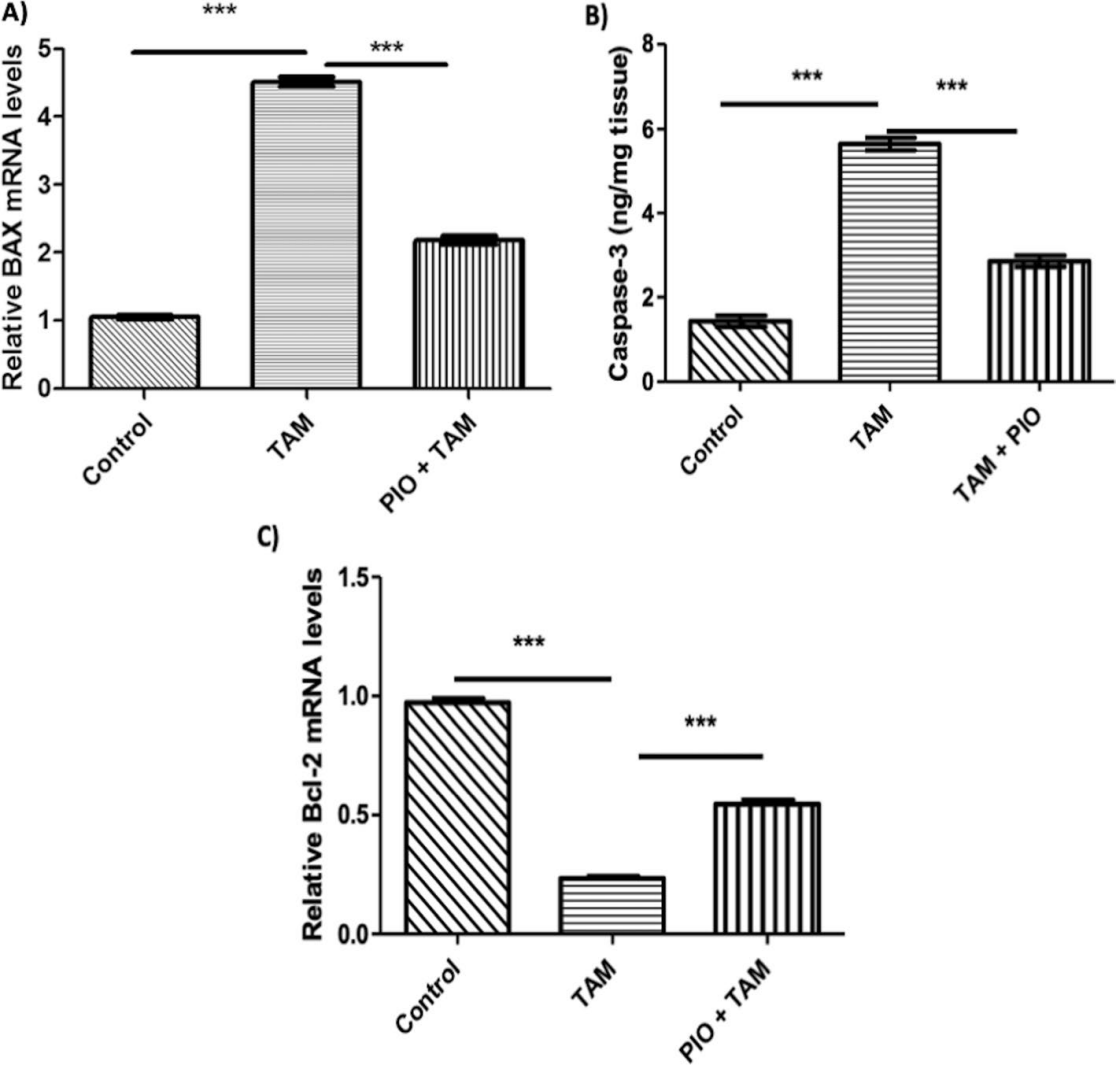
PIO prevented hepatic apoptosis in TAM-administered rats. PIO reduced hepatic Bax mRNA (**A**) and caspase-3 level (**B**) and raised hepatic Bcl-2 mRNA (**C**). Data are mean ± SEM, n = 6. **P < 0.01, and ***P < 0.001. *TAM* tamoxifen, *PIO* pioglitazone, *Bax* Bcl-2-associated X protein B-cell lymphoma, *Bcl2* B-cell lymphoma 2

To evaluate the protective impact of PIO on TAM-induced apoptosis, the expression levels of Bax, caspase-3, and Bcl-2 were evaluated. *TAM-*administered rats revealed marked upregulation of Bax (Fig. 5A) expression and caspase-3 level (Fig. 5B) whereas Bcl-2 (Fig. 5C) was downregulated (*P* < 0.001). However, PIO treatment was effective in ameliorating the expression level of caspase-3 and Bax levels and raising Bcl-2 mRNA.

### PIO treatment modulates hepatic Keap1/Nrf2/HO-1 signaling in TAM-received rats

**Fig. 6:**
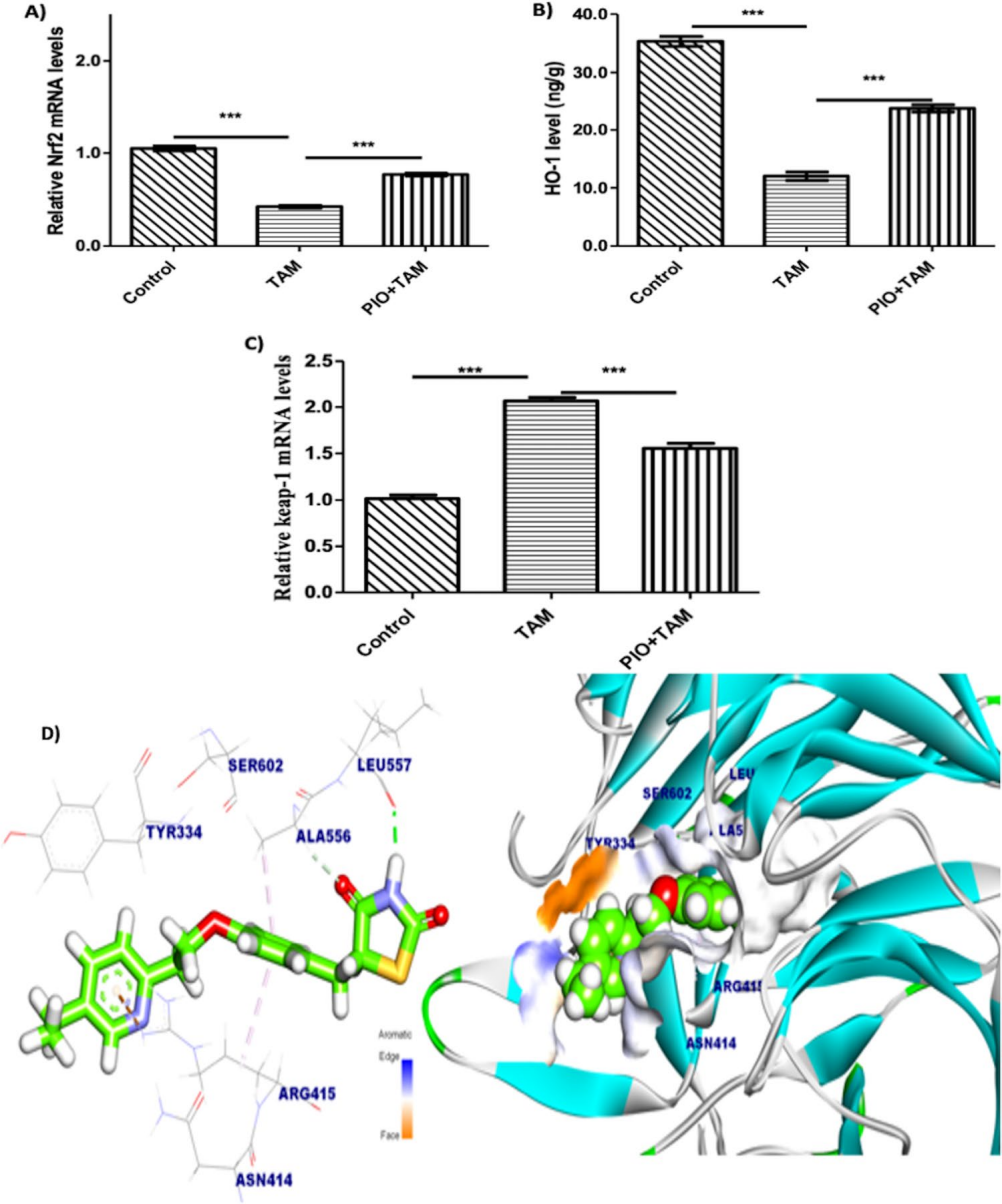
PIO upregulated renal Keap1/Nrf2/HO-1 signaling in TAM- received rats. PIO upregulated Nrf2 mRNA (**A**), increased HO-1 level (**B**), and reduced Keap1 mRNA (**C**) in TAM- received rats. Data are mean ± SEM, n = 6. *P < 0.05, **P < 0.01, and ***P < 0.001. **D** PIO and Keap1 interacted with each other multiple times, according to a molecular docking study. Three Pi-cation and Pi–Alkyl interactions with Ala556 and Arg415 as well as one hydrogen bond with the residue Leu557 are among these interactions. *TAM* tamoxifen, *PIO* pioglitazone, *Nrf2* nuclear factor erythroid 2-related factor 2, *HO-1* heme oxygenase-1, *Keap1* Kelch-like ECH-associated protein 1

To assess the impacts of TAM and PIO on Keap1/Nrf2/HO-1 signaling, we identified the mRNA abundance of Keap1, Nrf2, and HO-1 content. Additionally, we used molecular docking modeling to ascertain how PIO binds to Keap1. mRNA abundance of Nrf2 (Fig. 6A) and. HO-1 levels (Fig. 6B) were reduced whereas Keap1 mRNA (Fig. 6C) was elevated in the liver of TAM-received rats (*P* < 0.001). Contrarily, PIO treatment upregulated Nrf2 and downregulated Keap1 markedly compared to TAM-treated rats. As displayed in Fig. 6D, PIO was displayed to the dock with Keap1 formed three Pi–cation and Pi–Alkyl interactions with Ala556 and Arg415, forming one hydrogen bond with the residue Leu557 with a bond length of 2.15 Å. A potent binding to Keap1 for PIO was assessed depending on the scoring function value (− 7.65 kcal/mol).

### PIO treatment regulates hepatic SIRT1/Notch1 signaling in TAM- received rats

**Fig. 7:**
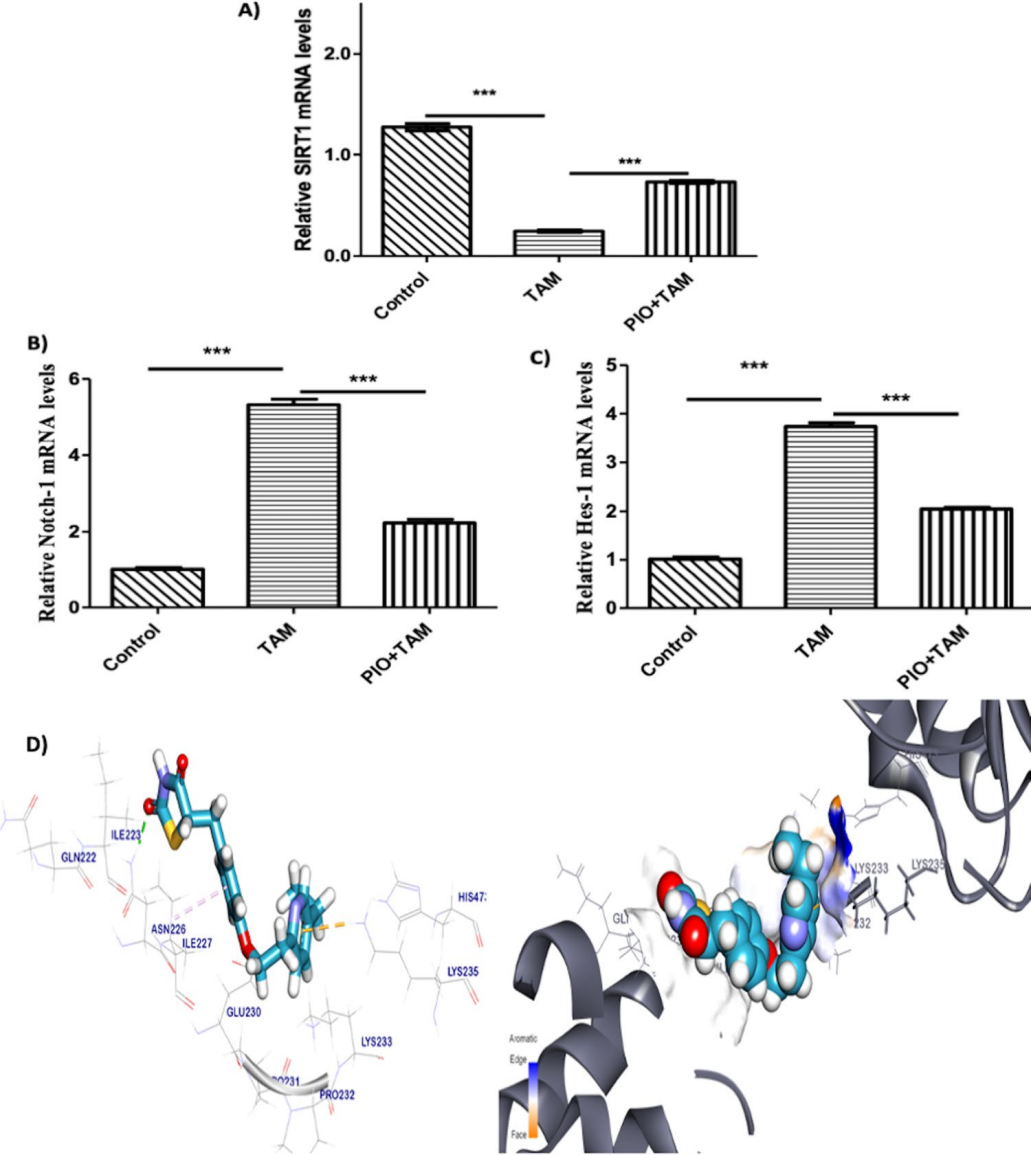
PIO upregulated hepatic SIRT1/Notch1 signaling in TAM- received rats. PIO upregulated SIRT1mRNA (**A**) and decreased Notch1 mRNA (**B**) along with reduced HES1 level (**C**) in TAM-received rats. Data are mean ± SEM, n = 6. *P < 0.05, **P < 0.01, and ***P < 0.001. **D** PIO and SIRT1 interacted with one another multiple times, according to a molecular docking study. In addition to a hydrogen bond, these interactions also involve the residues Ile227 and Lys235. *TAM* tamoxifen, *PIO* pioglitazone, *SIRT1* Sirtuin 1, *HES1* hairy and enhancer of split1

**Fig. 8:**
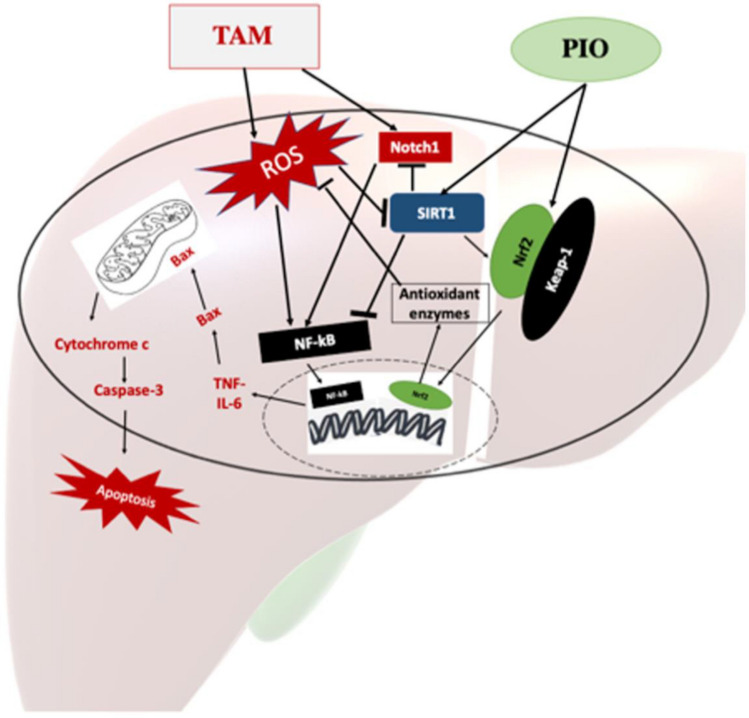
An illustration showing how PIO protects the liver from the toxicity of TAM. PIO prevented TAM-induced liver damage by regulating Nrf2/HO-1 and SIRT1/Notch1 signaling and mitigating oxidative stress, inflammation, and apoptosis

To assess the impacts of TAM and PIO on SIRT1/Notch1 signaling, we identified the mRNA abundance of SIRT1, Notch1, and Hes1 levels. Additionally, we used a molecular docking simulation to find out how PIO binds to SIRT1.TAM administration significantly decreased the expression of hepatic SIRT1 (Fig. 7A) while increasing the expression of Notch1 (Fig. 7B) and Hes1 (Fig. 7C) (*P* < 0.001). Co-treatment with PIO significantly upregulated SIRT1 and downregulated Notch1/Hes1 as compared to TAM-administered rats. Molecular docking was used to further investigate the positive impact of PIO on SIRT1, as seen in Fig. 8D. PIO’s molecular docking score revealed a binding energy of 7.73 kcal/mol, demonstrating compatibility with the SIRT1 active site. SIRT1 interacts with two amino acid residues, Ile227 and Lys235, via Pi–Alkyl and Pi–cation interactions and forms a hydrogen bond with Asn226 with a bond length of 2.29 Å.

## Discussion

Tamoxifen, an anti-estrogen non-steroidal agent, is frequently utilized as a chemotherapy medication to treat breast cancer [7]. Despite TAM’s broad therapeutic clinical applications, it exerts side effects, mainly on non-target organs. It is highly toxic for hepatic tissue than for any other tissue. Numerous reports of TAM side effects on the liver have been clarified in humans, involving steatohepatitis, hepatic steatosis, hepatic cirrhosis, and necrosis. Experimental investigations indicated that inflammation and oxidative stress are essential processes by which TAM stimulates inflammatory tissue damage [2, 10]. PIO is a crucial micronutrient having anti-inflammatory and antioxidant characteristics that also has a modulating influence on the apoptosis [32, 37, 38]. The aim of the current research was to investigate how PIO protects rats’ livers from TAM-induced hepatotoxicity.

In the present study, the administration of TAM (45 mg/kg) induced hepatotoxicity as evidenced by elevation in the levels of serum ALT, AST, and total bilirubin. Since they are found in the liver cytoplasm and are released into circulation as a consequence of cell injury, these results would suggest that TAM interferes with the structural integrity of liver tissues [39, 40]. Histological analysis further confirmed the TAM-induced liver damage, as demonstrated by a thickened hepatic capsule, extensive hepatocellular vacuolation, and portal fibroplasia with mononuclear inflammatory cell infiltration. TAM toxicity, which triggers inflammation, may be accountable for the invasion of inflammatory cells [41]. The shift in indices relating to liver function and the TAM-induced histological alterations were both significantly decreased by PIO co-treatment. These findings explore PIO’s preventive role against TAM-induced hepatotoxicity. Several investigations have documented the preventive role of PIO against experimentally drug-induced liver damage in this situation [28, 29, 38].

TAM toxicity is linked to ROS overload, inflammatory reaction, and oxidative injury [3, 10]. Various researches have shown that TAM may cause an imbalance in the redox status and induce oxidative liver damage [3, 9, 42, 43]. In this study, TAM administration significantly increased lipid peroxidation as evidenced by elevated MDA levels. In addition, TAM declined antioxidant defense mechanism as proved by the significant decline in hepatic SOD and GPx activity as well as the decreased GSH levels. It has been reported that anticancer therapy elicits free radical production [44]. TAM has been documented to impair the antioxidant/oxidative balance via exerting ROS production. An impact might be due to impaired beta-oxidation of fatty acids. Moreover, TAM has been reported to uncouple the mitochondrial electron transport chain, which leads to mitochondrial oxidative damage [3]. Concurrent administration of PIO showed a significant improvement in the oxidative status by elevation of antioxidant enzymes and depleting MDA levels. These findings agree with earlier research in different animal models [25, 28, 45]. Soliman et al. 2019 noted that PIO increased the expression of SOD and declined the level of MDA in the chronic kidney disease [46]. Moreover, It was hypothesized that the antioxidant effect of PIO was related to the PPAR-γ-dependent activity [47, 48].

ROS regulates gene expression of NF-κB, which is a spur of proinflammatory cascades via the liberation of pro-inflammatory mediators [13, 14]. Moreover, cytokines exacerbate inflammatory cell infiltration and increase oxidative stress. In this study, TAM triggered hepatic inflammation indicated by upregulated NF-κB p65 mRNA and elevated levels of pro-inflammatory cytokines (IL-6 and TNF-α). In the same context, TAM was documented to exhibit a marked increase in NF-κB protein expression along with IL-6 and TNF-α levels in rats [5, 7, 9]. Contrarily, PIO treatment downregulated NF-κB p65 mRNA expression and decreased pro-inflammatory cytokines (IL-6 and TNF-α). Previous reports indicated that PIO mitigated ischemia–reperfusion damage through preventing the expression of NF-κB related protein pathways [23, 49].

Besides oxidative stress and inflammation, TAM administration triggers the apoptotic pathway revealed by the diminished Bcl-2 and elevated caspase-3 and Bax in the liver tissues. These results agreed with prior investigations exhibiting TAM-induced apoptosis [3]. Bax is activated by excess ROS and pro-inflammatory cytokines, and this causes DNA damage and depletion of mitochondrial membrane potential, which together cause apoptosis [50]. In turn, caspase-3 activated and provoked cellular DNA fragmentation, and breakdown of cytoskeletal and cellular proteins, thus inducing cell death [51]. In contrast, PIO treatment significantly decreased apoptotic Bax expression and caspase-3 level along with increased antiapoptotic Bcl-2. These findings are in context with prior studies that displayed the antiapoptotic impact of PIO [15, 27, 52].

We presumed that regulation of Keap1/Nrf2/HO-1 and SIRT1/Notch signaling are involved in the hepatoprotective effectiveness of PIO. In this work, TAM reduced mRNA expression of Nrf2, and HO-1 content while Keap-1 mRNA increased, indicating ROS accumulation. Nrf2 knockdown has been documented under circumstances of exaggerated ROS caused by chemotherapeutic agents [12, 13, 53, 54]. In contrast, PIO treatment increased hepatic Nrf2/HO-1 signaling in TAM-treated rats. Previous reports have concluded that PIO exerts a protective effect through the regulation of the Nrf2 signaling [2, 28, 33]. Zakaria et al., 2019 reported that the anti-inflammatory actions of PIO-mediated NF-κB may be connected to Nrf2-reduced oxidative molecules necessary for its activation [55].

Furthermore, PIO administration elevated the expression level of SIRT1 and decreased Notch 1 in the liver of TAM-treated rats. Zhang et al., 2016 have documented that PIO activates SIRT1 expression in the model of cisplatin-induced nephrotoxicity in rats [56]. Contrarily, TAM administration caused a significant upregulation of Notch1, while the SIRT1 was downregulated. Prior investigation has verified a reduction in SIRT1 expression in the model of TAM-induced hepatic steatosis in high-fat-fed rats [57]. Moreover, TAM administration showed increased expression of Notch1 which is documented to upregulate NF‑κB transcriptional activity, in turn activating the inflammatory cascade and caspases [58]. Thus, inhibition of Notch1 expression has a vital role in reducing tissue inflammation [59–61]. Notch1 signaling has been stated as a downstream effector pathway of SIRT1 to affect disorder progressions, such as liver fibrosis [62]. SIRT1 may negatively control the Notch1 signaling pathway by deacetylating NICD, which interrupts the acetylation-induced stability of NICD, needed for the Notch1 activation [63]. These findings indicate that PIO modulated SIRT1/Notch1 expression and which in turn, ameliorated TAM-induced inflammation. Likewise, SIRT1/Notch1 modulation was earlier stated to improve inflammation and oxidative stress in the model of diabetic retinopathy in rats [62]. SIRT1 activation can regulate ROS production, enhance mitochondrial function, and ceases cellular oxidative stress [64]. Drugs that stimulate SIRT1 reduce TNF-α-induced NF-κB activation, which is brought on by deacetylation of the p65 protein [65]. Moreover, the SIRT1 activation up-regulates the Nrf2/HO-1 signaling pathway, an effect that was induced via the deacetylation of the Nrf2 [20].

We carried out a molecular docking investigation to learn more about PIO’s hepatoprotective process and to learn how it binds to Keap1, NF-κB, and SIRT1. According to our findings, Keap1 and PIO may interact, and PIO may also occupy the Nrf2 site of action. PIO’s measured energy binding affinity for Keap1 is − 7.65 kcal/mol, which suggests that PIO most likely inhibits Keap1. The beneficial effect of PIO may be brought about through its interaction with Keap1 and the inhibition of Keap1’s binding to Nrf2, which encourages the transcriptional production of the antioxidative genes. The prior described PIO-induced SIRT1 activation via multiple mechanisms is noteworthy. Our in-silico research revealed the binding patterns and interaction processes of SIRT1 with PIO. Due to the hydrogen bonds, hydrophobic contacts, and enough binding affinity, the molecular docking assessment showed that PIO and SIRT1 had a strong binding affinity. PIO demonstrated a binding affinity for NF-κB as well as Keap1 and SIRT1. Due to PIO’s ability to block the NF-κB signaling pathway, the in silico docking model of PIO with the NF-κB complex demonstrated that PIO had a substantial binding affinity.

## Conclusions

This work demonstrated how PIO modifies SIRT1/Notch1 and Keap1/Nrf2/HO-1 signaling to safeguard against TAM-induced hepatotoxicity. PIO reduced liver damage decreased ROS and the pro-inflammatory response, and attenuated apoptosis in addition to lowering the levels of circulating liver function biomarkers. Along with these outcomes, SIRT1 and Nrf2/HO-1 signaling were stimulated, NF-κB and Notch1 were downregulated, and antioxidant defenses were strengthened. The in-silico docking modeling suggested that PIO might bind to NF-κB, Keap1, and SIRT1 with some activity. Therefore, PIO’s capacity to reduce oxidative stress, inflammation, and alter different signaling might act as a protective mechanism against TAM-induced hepatotoxicity in medical environments. Nevertheless, further research and clinical trials are needed to fully understand its therapeutic activity (s).

## Data Availability

The manuscript contains all data supporting the reported results.
